# Synthesis and Investigation of Vanadium-Based Catalysts for the Oxidation of 4-Methylpyridine to Isonicotinic Acid

**DOI:** 10.3390/ijms27062715

**Published:** 2026-03-16

**Authors:** Nurdaulet Buzayev, Kairat Kadirbekov, Mels Oshakbayev

**Affiliations:** 1 A.B. Bekturov Institute of Chemical Sciences, Almaty 050010, Kazakhstan; o_mels@mail.ru; 2 Department of Physical Chemistry, Catalysis and Petrochemistry, Al-Farabi Kazakh National University, Almaty 050040, Kazakhstan

**Keywords:** catalyst, oxidation, isonicotinic acid, 4-methylpyridine, green technology, synthesis, petrochemical by-product

## Abstract

The study investigates the catalytic activity of vanadium-containing catalysts in the selective oxidation of 4-methylpyridine (4-MP) in the gas phase. V-Cr, V-Ti, and V-Ti-Cr catalysts were synthesised and studied. The phase composition and structural features of the catalysts were determined by X-ray diffraction (XRD) and Raman spectroscopy, and their thermal stability was investigated using thermogravimetric analysis (TGA/DTA). Textural characteristics were evaluated by low-temperature nitrogen adsorption–desorption (BET, BJH), surface morphology was studied using scanning electron microscopy (SEM), and the distribution of elements was investigated using energy-dispersive X-ray spectroscopy (EDX). The chemical composition of the catalysts was determined using inductively coupled plasma atomic emission spectrometry (ICP-OES) and catalytic activity was evaluated in the selective gas-phase oxidation reaction of 4-methylpyridine in the temperature range 280–380 °C. It was found that an increase in temperature is accompanied by an increase in the conversion of 4-methylpyridine, but at the same time, deep oxidation reactions intensify. The best result is achieved on the V-Ti-Cr catalyst, for which the conversion of 4-MP reaches 86.88% and the selectivity is 73.06% at 320 °C. However, V-Ti provides moderate stable performance, while V-Cr demonstrates relatively low efficiency. Thus, it can be concluded that the nature of the temperature dependence of 4-methylpyridine conversion reflects the different nature of the active centres and their stability.

## 1. Introduction

Today, tuberculosis (TB) remains a serious public health problem. According to statistics, one in four people on the planet is infected, and without treatment, 5–10% of them develop an active form of the disease [1]. Classic pulmonary tuberculosis manifests itself in the form of a prolonged cough (lasting three weeks or more), sometimes with blood, as well as low-grade fever, night sweats, significant weight loss, and general weakness [2,3,4,5]. Tuberculosis mainly affects the young and working-age population (15–49 years) and carries a high social burden. The global direct costs of diagnosing and treating tuberculosis exceed $6 billion per year, and the indirect costs of lost productivity and reduced productivity are estimated to be even higher [6,7]. According to World Health Organization (WHO) estimates, in 2023, the incidence of tuberculosis worldwide was ≈10.8 million people (134 cases per 100,000), which is higher than 2022 (10.7 million) and 2021 (10.4 million) [6,8,9]. The number of deaths from tuberculosis was approximately 1.25 million. The WHO indicates that tuberculosis has once again become the leading cause of death from infectious diseases [5,6,8].

The main drugs used in the treatment of tuberculosis are isoniazid, rifampicin, ethambutol, and pyrazinamide, which are prescribed for several months (6–9 months) [10,11]. Isoniazid (isonicotinic acid hydrazide, INH) is a synthetic antibiotic and one of the most effective and important drugs for the treatment of tuberculosis [12]. Not only isoniazid but also other important anti-tuberculosis drugs, such as tubazid, ftivazid and saluzid, are synthesised from isonicotinic acid (INA) and its derivatives [13]. The production of these compounds is based on the oxidative treatment of 4-methylpyridine, which is first isolated from the light pyridine base fraction as a mixture of methylol derivatives. The main source of γ-picoline (4-methylpyridine, C_6_H_7_N) is petrochemical by-products, in particular, crotonic and acetylene fractions containing mixtures of pyridine bases [14,15].

Isonicotinic acid can be obtained by two main existing catalytic methods: oxidation of γ-picoline either in the liquid phase (using aqueous oxidants such as HNO_3_, H_2_O_2_ or pressurised air with Co/Mn catalysts) or in the gas phase (using O_2_ or air over metal oxide catalysts at high temperature) [16,17,18]. The liquid phase method has undeniable advantages such as high selectivity of the target product formation, as well as easy availability and low cost of oxidants. However, due to the fact that the method is multi-stage and involves the use of strong acids, which is associated with the formation of hazardous and toxic salt and acid waste and leads to the need for disposal and the use of expensive anti-corrosion equipment, this process cannot be considered environmentally friendly or ‘green’ [19,20,21].

Oxidative ammonolysis of 4-methylpyridine can lead to the formation of isonicotinic acid nitrile [20,22], the hydrolysis of which allows the synthesis of isonicotinic acid. Despite its widespread practical application, this route is considered technologically and environmentally imperfect for several reasons:

- Increased requirements for explosion safety and corrosion resistance of equipment due to the need for simultaneous supply of NH_3_ and O_2_ at 350–450 °C [23].
- Subsequent hydrolysis of 4-cyanopyridine proceeds under harsh conditions, accompanied by the formation of by-products, a decrease in selectivity, and complications in purification to regulatory requirements [24].
- The multi-stage nature of the process increases reagent consumption, waste volume and energy costs, and scaling is limited by problems with thermal conditions, catalyst stability and reduced selectivity [25,26].

Gas-phase methods are being developed as an alternative. In particular, the oxidation reaction of 4-MP to isonicotinic acid is possible in a single-stage process on heterogeneous catalysts with oxygen as an oxidant at atmospheric pressure in the presence of a small amount of water [23,27,28].



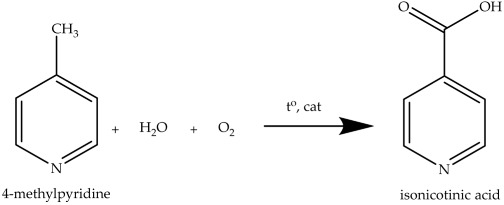

(1)


Important advantages of gas-phase oxidation are the use of air instead of a stoichiometric oxidant, high selectivity, and simplified technology with low waste levels [29]. For example, catalytic oxidation of 4-picoline on a catalyst of vanadium modified with titanium dioxide leads to the formation of target products (aldehyde and carboxylic acid) ‘in one step’ [30], which is fundamentally advantageous. All this has sparked considerable scientific interest in the gas-phase oxidation of γ-picoline, since improving this process directly affects the availability and cost of drugs based on isonicotinic acid. In practice, catalysts based on transition metal oxides are used for the gas-phase oxidation of 4-methylpyridine. Among these traditional catalysts, vanadium oxide-based systems dominate.

The study of the partial oxidation of 4-picoline on a catalyst made of individual oxide (V_2_O_5_) showed that this catalyst allows high catalytic activity: already at a temperature of 270, the conversion of 4-picoline is 77% [31]. Under these conditions, pyridine 4-aldehyde is formed with higher selectivity than isonicotinic acid (64.9% and 32.5%, respectively). However, as experimental data show [23,32], the use of pure V_2_O_5_ has a number of significant limitations. At low temperatures (250 °C), the conversion of 4-methylpyridine does not exceed 40%, and the yield of isonicotinic acid is only 4–9%. The main product is the intermediate product 4-pyridinecarbaldehyde, which indicates that the reaction stops at the partial oxidation stage. Further heating leads to an increase in the yield of isonicotinic acid from 27% (290 °C) to a maximum value of 58.6% (350 °C), but this is accompanied by an increase in the amount of side products of complete oxidation CO and CO_2_ (total yield 80%) [33].

Modifying V_2_O_5_ with additives of other oxides as a carrier (SnO_2_, ZrO_2_, Al_2_O_3_, etc.) is a way to increase activity and selectivity. A study of the partial oxidation of 4-picoline on a V-Ti-Al-O catalyst showed that at a temperature of 330 °C, the yield of 4-picoline is 54.3% and the selectivity of pyridine-4-aldehyde and isonicotinic acid formation (40% and 80%, respectively) demonstrates high catalytic activity [32]. V_2_O_5_-TiO_2_-based systems are one of the most widely studied catalysts for the oxidation of 4-methylpyridine. The addition of titanium (IV) oxide to vanadium oxide (V_2_O_5_) is traditionally considered a way to increase the activity and selectivity in alkylpyridine oxidation reactions [25,34,35]. Thus, in work [23], it is shown that binary V_2_O_5_-TiO_2_ systems have higher catalytic activity than pure V_2_O_5_. The conversion of 4-methylpyridine increases with reaction temperature from 230 °C to 310 °C, reaching a complete conversion (100%) and the yield of nicotinic acid hits 40%. Also, other studies [28] imply that the V_2_O_5_-rutile catalyst at a temperature of 330 °C attains a conversion of 40% and the maximum acid yield of 22%.

It is known that CrO_3_ is also an active catalyst for partial oxidation, but it tends to deeply oxidise organic compounds of various classes [34]. As shown in [29,34], the chemical compound CrVO_4_ is characterised by low activity and acts as a carrier. On the binary catalyst 2V_2_O_5_–Cr_2_O_3_, the maximum yield of isonicotinic acid is about 64% at sufficiently high temperatures (330 °C) [27]. The introduction of titanium oxide into this system (2V_2_O_5_·4TiO_2_·Cr_2_O_3_) leads to a transition to a low-temperature regime, where the maximum yield 67% of isonicotinic acid is achieved at a temperature of 250 °C with 100% conversion [27].

The above data on catalysts obtained by mechanical mixing demonstrate important patterns showing that the addition of TiO_2_ and/or Cr_2_O_3_ changes both the optimum temperature for isonicotinic acid (INA) yield and the maximum selectivity values. At the same time, there is a simultaneous increase in the probability of undesirable ‘deep oxidation’ when the temperature is exceeded or when the phase ratio is unfavourable [23,32]. These observations emphasise that further improvement of properties requires control of the microstructure, dispersion, and local chemistry of active centres—that is, those parameters that largely depend on the catalyst synthesis method. Experimental data show that the key characteristics of oxide catalysts are determined not only by their stoichiometry, but also by a combination of microstructural and surface parameters: the morphology and size of vanadium phase crystallites; the uniformity of vanadium and promoter distribution in the matrix; specific surface area and porosity; density and nature of oxygen vacancies and oxygen transfer channels; acid-base properties of the surface (Lewis/Brønsted centre ratio); as well as thermal and structural stability during long-term operation [36,37].

Based on the fact that the key factors for catalyst efficiency are the homogeneity of active centre distribution and control of phase composition at the microstructural level, the Pechini method was chosen for the synthesis of catalytic systems in this work. Unlike mechanical mixing of oxides, this approach, based on the formation of a polymer precursor, ensures the fixation of metal cations in the polymer network, preventing their premature phase separation. This allows for the mixing of components at the molecular level, which is critical for the formation of complex non-stoichiometric phases and mixed oxides responsible for catalytic activity [38,39,40,41,42,43,44,45]. Thus, the use of the Pechini method in this study is aimed at obtaining highly dispersed systems with a reproducible architecture of active centres and a developed surface, which is a necessary condition for increasing the selectivity of the partial oxidation of 4-methylpyridine.

In previous works [20,29], we investigated binary V-Ti and V-Cr systems, as well as a ternary V-Ti-Mn system, for which an increased specific surface area (up to 44.60 m^2^/g according to BET) was obtained. This raised a fundamental question: is the increase in textural characteristics a specific feature of manganese or is it a fundamental property of three-component systems obtained by the Pechini method? In addition, the joint behaviour of Ti and Cr in the V-Ti-Cr system has not been analysed to date. In this regard, the aim and novelty of this work was to establish the nature of the joint influence of Ti and Cr in the V-Ti-Cr system and to determine whether the transition to a new three-component configuration leads to a similar synergistic effect in the oxidation reaction of 4-methylpyridine.

## 2. Results and Discussion

### 2.1. Catalyst Structure Analysis

**Figure 1:**
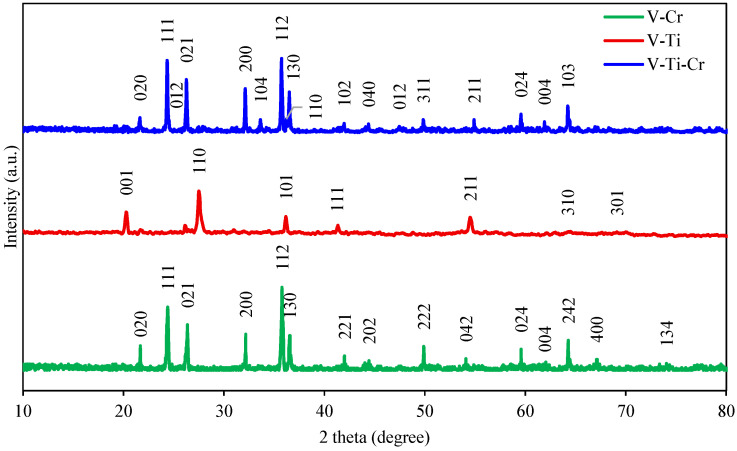
X-ray diffraction pattern of the catalysts.

The XRD pattern of the catalysts is shown in Figure 1. The peaks at 2θ ≈ 21°, 24°, 26°, 32°, 36°, 37°, 42°, 50°, 54°, 62°, 64°, 67° and 74°, which correspond to the crystallographic planes (020), (111), (021), (200), (112), (130), (221), (222), (042), (004), (242), (400) and (134), are in good agreement with the standard data for the orthorhombic phase of CrVO_4_ (PDF 84-1740). The peaks at 2θ = 44° and 60° correspond to the (202) and (024) planes of V_2_O_5_ (PDF 77-2415), while the diffraction peak at 2θ = 20° refers to the (001) plane of the orthorhombic phase of V_2_O_5_ (PDF 41-1426). The main matrix phase of the V-Ti catalyst is rutile TiO_2_ (PDF 21-1272) with peaks at 28°, 41° and 55° related to the (110), (111) and (211) planes, as well as TiVO_4_ (PDF 77-0332) with peaks at 36°, 64° and 69° attributed to the (101), (310) and (301) planes. Reflections at 25° (012), 34° (104), 36° (110) and 47° (012) are attributed to the Cr_2_O_3_ phase (PDF 38-1479). The peak at 2θ ≈ 50° corresponds to the (311) reflection of the mixed Cr_2_TiO_5_ (PDF 35-0781) pseudorutite-type phase, indicating the interaction of chromium with TiO_2_. The peaks at 2θ ≈ 42° (102) and 64° (103) are attributed to the non-stoichiometric phase Cr_0_._11_V_2_O_5_._16_, which indicates partial isomorphic substitution of vanadium by chromium ions in the vanadium oxide structure.

The observed differences in the diffraction patterns are due to chemical interaction between the active components (V, Cr) and the carrier (TiO_2_). The introduction of chromium into the V-Ti system leads to a significant decrease in the intensity of the free vanadium oxide peaks due to its binding in the highly crystalline orthorhombic phase of chromium vanadate (CrVO_4_). At the same time, the formation of a mixed phase of pseudorutile Cr_2_TiO_5_ indicates a partial interaction of chromium with the titanium dioxide matrix, which is also accompanied by a redistribution of the intensities of the rutile peaks. The detection of the Cr_0_._11_V_2_O_5_._16_ confirms the occurrence of isomorphous substitution of V^5+^ ions by Cr^3+^ ions, which causes local distortions of the crystal lattice and a shift in the diffraction maxima. Differences in the width and intensity of the peaks indicate a change in the dispersion of the phases. The V-Ti sample has high dispersion (broad peaks), while the introduction of Cr stimulates the growth of large CrVO_4_ and Cr_2_TiO_5_ crystallites, which manifests itself in the narrowing and increase in intensity of the diffraction peaks.

### 2.2. Vibrational Characterisation of Catalysts

**Figure 2:**
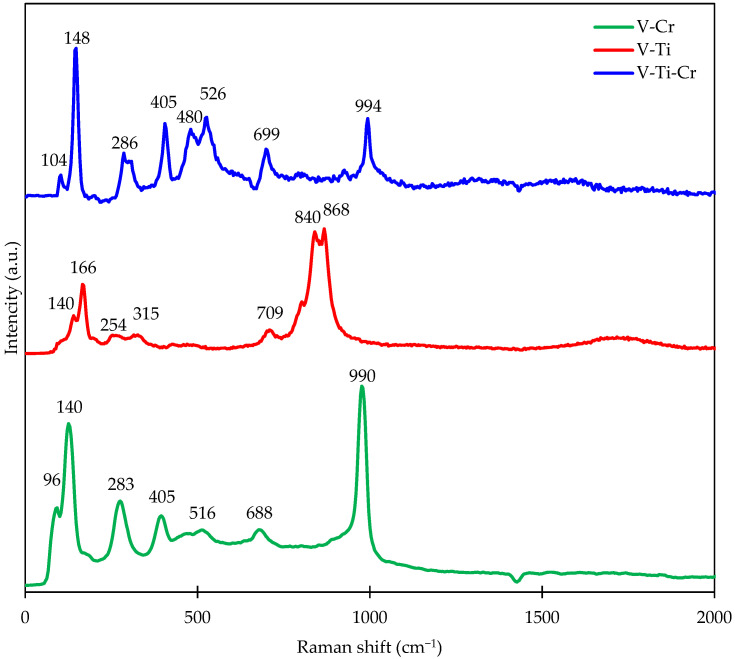
Raman spectrum of the catalysts.

The Raman spectrum of the V-Cr, V-Ti, and V-Ti-Cr catalytic systems are shown in Figure 2. The detailed distribution of bands for all catalysts is shown in Table 1. The Raman spectra show characteristic bands that can be correlated with certain vibrational modes of the crystal lattice and vanadium-oxygen network, as confirmed by the literature data [46,47,48,49,50,51,52]. In the low frequency range (~104–148 cm^−1^), bands corresponding to lattice translational motions and B_1_g vibrations are observed, reflecting the rigidity and connectivity of the oxide lattice, with the replacement of vanadium ions by Ti or Cr leading to a shift in these bands. In the range 140–148 cm^−1^, bending and rotational vibrations of the V-O lattice are recorded, associated with the deformation of V-O-V bonds and the influence of introduced Ti or Cr ions. The bands at 283–286 cm^−1^ correspond to O-V-O bending, while the band at 315 cm^−1^ reflects internal V-O vibrations in octahedral fragments that are sensitive to the local coordination of vanadium atoms. The band at 405 cm^−1^ is observed in V-Ti and V-Ti-Cr systems and is associated with deformation modes of the VOx skeleton, reflecting the connectivity of the vanadium-oxygen network. The bands at 480 cm^−1^ (V-O-V in the ribs) and 516–526 cm^−1^ (B_1_g + Ag, bending and stretching of O-V-O bridges) demonstrate the influence of oxygen bridge bonds and the degree of polymerisation of the VOx network, with their shift indicating partial replacement of V by Ti or Cr. In the 688–699 cm^−1^ region, internal V-O stretches are observed, and the broad band at 840–868 cm^−1^ in the V-Ti system corresponds to network stretches associated with terminal and bridging V-O bonds. The combination of Raman spectroscopy and XRD data allows us to conclude that vanadium in the V-Ti sample is present in two forms: as finely dispersed surface complexes and as a small amount of the bulk crystalline phase V_2_O_5_, whose diffraction maxima were recorded by X-ray structural analysis. At the same time, the absence in the Raman spectrum of a dominant peak at 994 cm^−1^, characteristic of large-crystalline V_2_O_5_, indicates a high degree of dispersion and defectiveness of the vanadium oxide crystal lattice on the surface of TiO_2_. In V-Cr systems, the band at 990 cm^−1^ corresponds to symmetric Cr-O Cr(VI) type stretches, and the terminal V=O bond appears at 994 cm^−1^ in V-Ti-Cr, indicating the presence of highly oxidised vanadium phases, which corresponds to X-ray diffraction reflections, V_2_O_5_ and Cr_0_._11_V_2_O_5_._16_. In general, the observed modes and their shifts confirm the formation of mixed V-Ti-Cr oxides with octahedral and tetrahedral VOx fragments, as well as the possible participation of V-O-Cr bridge bonds, which are consistent with the X-ray diffraction analysis data for CrVO_4_ (PDF 84-1740).

### 2.3. Thermal Properties of Synthesised Catalysts

**Figure 3:**
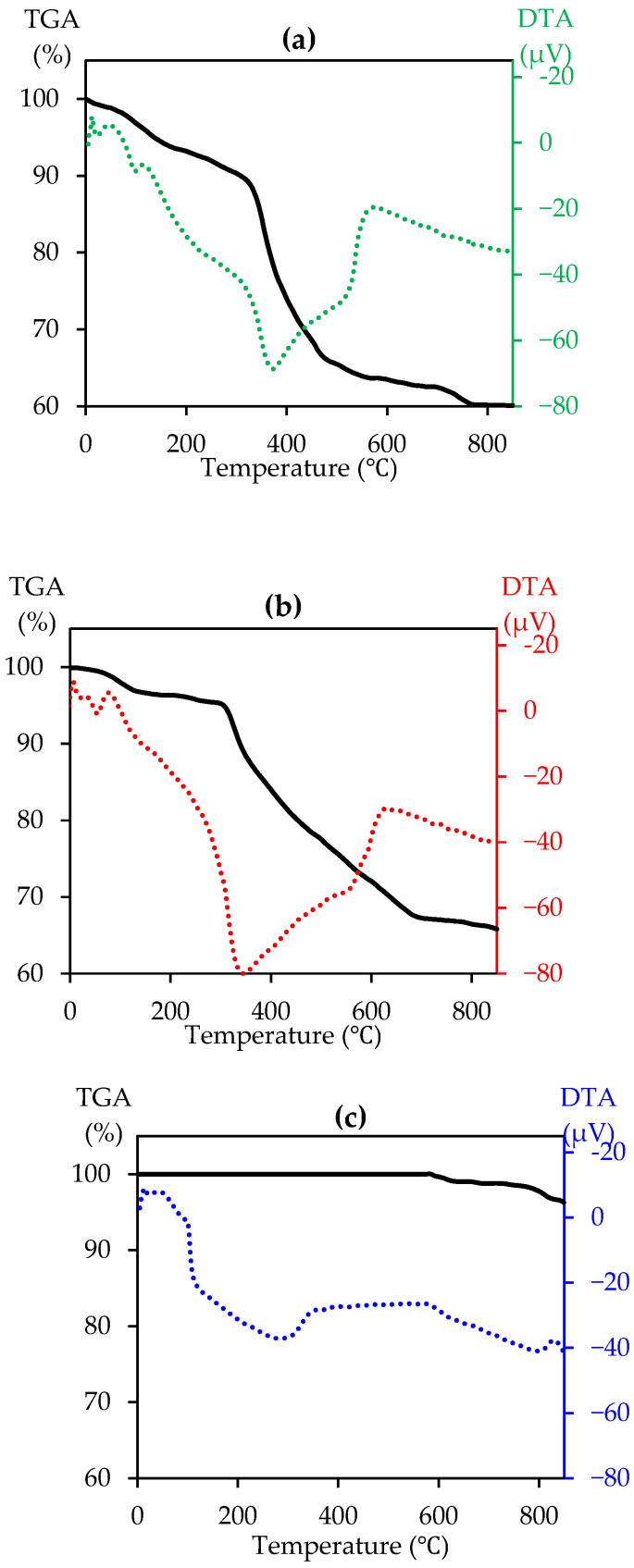
TGA and DTA of the catalysts: (**a**) V-Cr; (**b**) V-Ti; (**c**) V-Ti-Cr.

For V-Cr and V-Ti catalysts (Figure 3a,b), slow heating to ≈100 °C results in a small mass loss of about 5%, accompanied by weak differential thermal analysis (DTA) reactions, which is interpreted as the removal of physically adsorbed water and volatile impurities after synthesis. In addition, in the range of ≈100–350 °C, thermogravimetric analysis (TGA) shows a relatively modest decrease in mass to approximately ≈90% (V-Cr) and 95% (V-Ti), while DTA shows an abrupt thermal effect (with a signal intensity up to −70–80 μV). Above 350 °C, the mass curve drops sharply to ≈66–67% at ≈500 °C (V-Cr) and 700 °C (V-Ti). These features of the thermal profile correspond to the phases detected by X-ray diffraction of rutile TiO_2_ (PDF 21-1272), Cr_2_O_3_ (PDF 38-1479), and partially formed mixed oxides CrVO_4_ (PDF 84-1740). For the V-Cr catalyst at temperatures above ≈450 °C, thermogravimetric analysis (TGA) transitions to a mode of gradual mass reduction to ≈60% at 850 °C. The DTA curve shows a return to a stable baseline (reaching approximately 10 μV) at the final stage of the analysis. Here, the appearance of a distinct Raman band at around 990 cm^−1^, corresponds to terminal vanadium bonds V=O, while bands in the range 400–700 cm^−1^ characteristic of bridge bonds V-O-Cr are simultaneously amplified, indicating the formation of highly oxidised vanadium centres and the beginning of the formation of a mixed oxide phase of the V-Cr type. For V-Ti, this effect is observed after 600 °C, and the mass loss stabilises at less than 2%, consistent with the preservation of bands characteristic of the rutile phase of TiO_2_ (≈140, 166, 254, and 315 cm^−1^), as well as with the presence of V-O-Ti bonds identified in the Raman spectrum, indicating a weakening of rapid chemical transformations and the predominance of slow processes of crystallite coarsening.

The thermogravimetric and differential thermal curves of the V-Ti-Cr sample (Figure 3c) show a pattern that differs significantly from the other two systems. No mass loss is recorded up to 600 °C. At the same time, in the range of ~150–350 °C, there is an endothermic maximum on the DTA, which should be interpreted as the process of crystallisation of the amorphous phase and the formation of primary crystalline nuclei of mixed oxides CrVO_4_ and Cr_2_TiO_5_. This process is accompanied by final dehydroxylation of the surface and the formation of strong Me-O-Me bonds. In the rank of 350–650 °C, DTA retains relatively moderate negative values with local fluctuations, which corresponds to a gradual reorganisation of the oxide lattice. This conclusion is confirmed by the intensification of characteristic V-O-Ti bridge bonds, which indicates the stabilisation of polyvanadate structures in the presence of titanium. The parallel presence of chromium is manifested in the appearance of weak but stable Cr-O vibration bands, reflecting the formation of distributed CrVO_4_-type clusters. The coincidence of thermoanalytical and spectroscopic data confirms that it is in the range of 400–600 °C that the fixation of mixed Ti-Cr-V oxide domains, which ensure the catalytic stability of the system, is completed. From 600 to 900 °C, only a slight mass loss of about 5% is observed, indicating the onset of irreversible processes (sintering, transition to more stable but less dispersed phases).

### 2.4. Catalysts Surface and Pore Properties

The textural properties of the catalysts were studied by low-temperature nitrogen adsorption–desorption using BET and BJH models (Table 2). The results obtained indicate a significant influence of the component composition on the development of the specific surface area and porous structure of the catalysts. The V-Cr and V Ti catalysts have an extremely low specific surface area (1.378 and 2.047 m^2^/g), specific surface area according to the BJH method (0.9437 and 0.7539 m^2^/g) and insignificant pore volume according to the BJH method (=0.000678 and 0.000384 cm^3^/g), which indicate the formation of a dense, weakly porous structure. Thermogravimetric analysis/differential thermal analysis for these systems recorded pronounced endothermic peaks and sharp sections of structural rearrangement, indicating local overheating and intense sintering of particles. These phenomena led to the destruction of the mesoporous structure, and X-ray diffraction analysis data confirmed the formation of large crystallites and separate oxide phases of Cr_2_O_3_ and TiO_2_ with a high degree of order. Raman spectra also recorded a predominance of crystalline oxides and a decrease in the proportion of distributed VOx fragments, since the agglomeration of vanadium-containing clusters reduces the availability of active centres and destroys the fine-grained structure. V-Ti-Cr exhibits a highly developed texture with a BET specific surface area of 57.5891 m^2^/g and a BJH specific surface area of 66.4401 m^2^/g. The significant discrepancy between the BET and BJH areas indicates a well-developed mesoporous structure that provides high surface accessibility for reagent molecules. The pore volume of this catalyst (0.035257 cm^3^/g) is two orders of magnitude higher than that of binary samples, confirming the formation of a branched porous network. Such a sharp (more than 25-fold) increase in specific surface area in the V-Ti-Cr sample compared to V-Ti and V-Cr indicates the presence of a pronounced synergistic effect. In binary systems, the absence of obstacles to the diffusion of homogeneous particles leads to intensive sintering and the formation of dense structures with low porosity. However, in a trimetallic system, chromium, titanium, and vanadium act as mutual structural promoters (disconnectors). The joint presence of three phases and the formation of mixed compounds (CrVO_4_, Cr_2_TiO_5_) limit the migration and aggregation of individual oxide grains during calcination. Thus, the components of the system mutually block each other’s tendency to crystallisation growth, which allows maintaining a highly developed mesoporous structure and ensuring a large pore volume. This confirms that the introduction of chromium into the V-Ti system not only adds a new active centre, but also fundamentally changes the architecture of the catalyst, preventing the collapse of pores characteristic of binary oxides.

**Figure 4:**
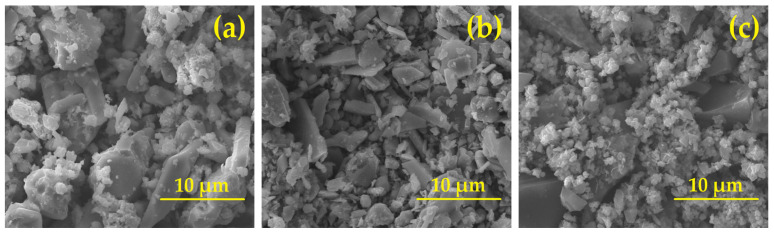
SEM of the vanadium-containing catalysts: (**a**) V-Cr; (**b**) V-Ti; (**c**) V-Ti-Cr.

The morphology of the catalyst samples was studied using SEM, and the results are shown in Figure 4. The SEM image of V-Cr (Figure 4a) shows a pronounced polydisperse structure of the sample. The morphology is represented mainly by angular and lamellar particles of irregular shape with sizes of about 1–8 μm. The surface of large fragments is partially covered with a fine-grained phase. A high degree of aggregation is noted. The surface of the particles is rough, with microdefects and chips. Thus, SEM data supports the weak textural development of the V-Cr catalyst. V-Ti (Figure 4b) is characterised by a more pronounced lamellar morphology. The particles are elongated with relatively clear edges and sizes in the range of ~2–10 μm. Compared to V-Cr, a more ordered crystalline texture is observed. The fine-grained phase is less prevalent and is mainly located in the intergranular space. The surface of the particles is less porous, with smoother areas. The data obtained morphologically justify a moderate increase in S_BET_ from 1.3780 to 2.0470 m^2^/g compared to V-Cr but confirm the preservation of the predominantly dense and low-porosity structure of the V-Ti catalyst. The morphology of the V-Ti-Cr catalyst (Figure 4c) reveals a significant increase in the proportion of the finely dispersed phase. The surface of large particles is almost completely covered with agglomerates of nanometric and submicron size. The structure is characterised by a high degree of looseness and a developed texture. There is a decrease in the average size of large fragments and an increase in the aggregation of small particles. Consequently, the SEM results are in good correlation with the adsorption measurements, demonstrating that the introduction of Ti and Cr leads to the formation of a highly dispersed texture and a significant increase in S_BET_ compared to binary systems. Overall, the results of SEM analysis confirm the BET measurement data and demonstrate a clear correlation between the morphology, degree of dispersion and textural characteristics of the catalysts under study: V-Cr < V-Ti < V-Ti-Cr.

**Figure 5:**
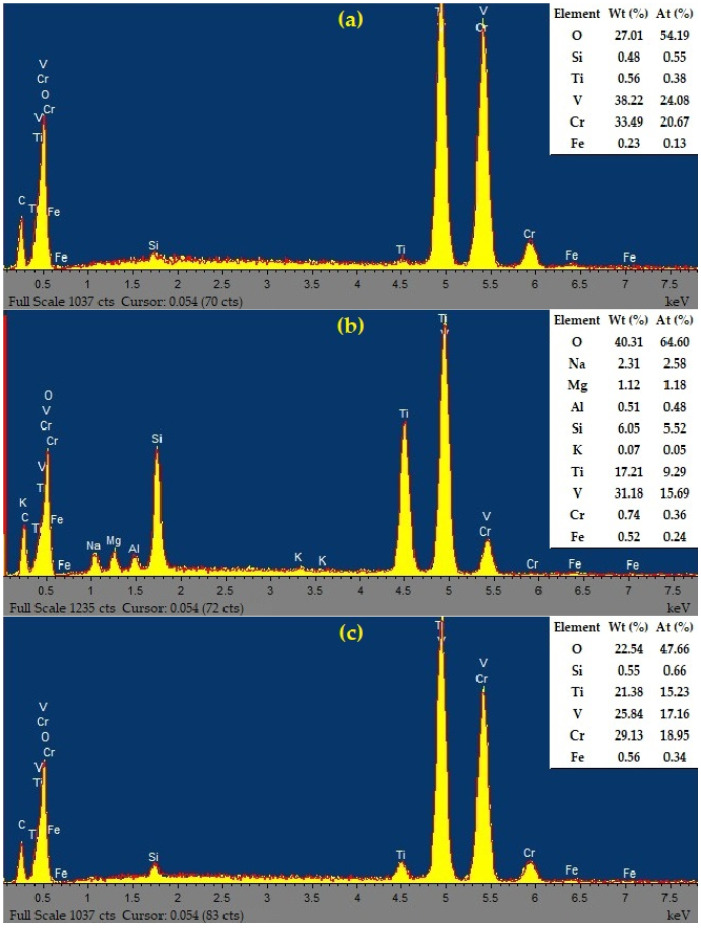
EDX spectra of synthesised catalysts: (**a**) V-Cr; (**b**) V-Ti; (**c**) V-Ti-Cr.

The results of EDX analysis of V-Cr, V-Ti, and V-Ti-Cr catalysts show the presence of all constituent elements in the final nanostructure (Figure 5). The presence of the main elements V, Cr and Ti with a high oxygen content confirms the formation of oxide phases. The presence of small amounts of Si, Fe and other elements indicates insignificant impurities, while the main metals are distributed close to the declared composition. Thus, EDX data confirm the successful introduction of the main metals into the oxide matrix while maintaining the declared proportions, despite the presence of insignificant impurities.

The actual content of the synthesised catalysts was determined using the ICP-OES method. Table 3 shows the stoichiometric ratio of vanadium (V), chromium (Cr) and titanium (Ti) in the V-Cr, V-Ti and V-Ti-Cr catalysts. The actual metal concentration values are close to aimed values, with small deviations in Cr and Ti in multicomponent catalysts probably due to partial losses during synthesis or incomplete incorporation of metals into the carrier.

### 2.5. Catalytic Performance of the Catalysts

**Figure 6:**
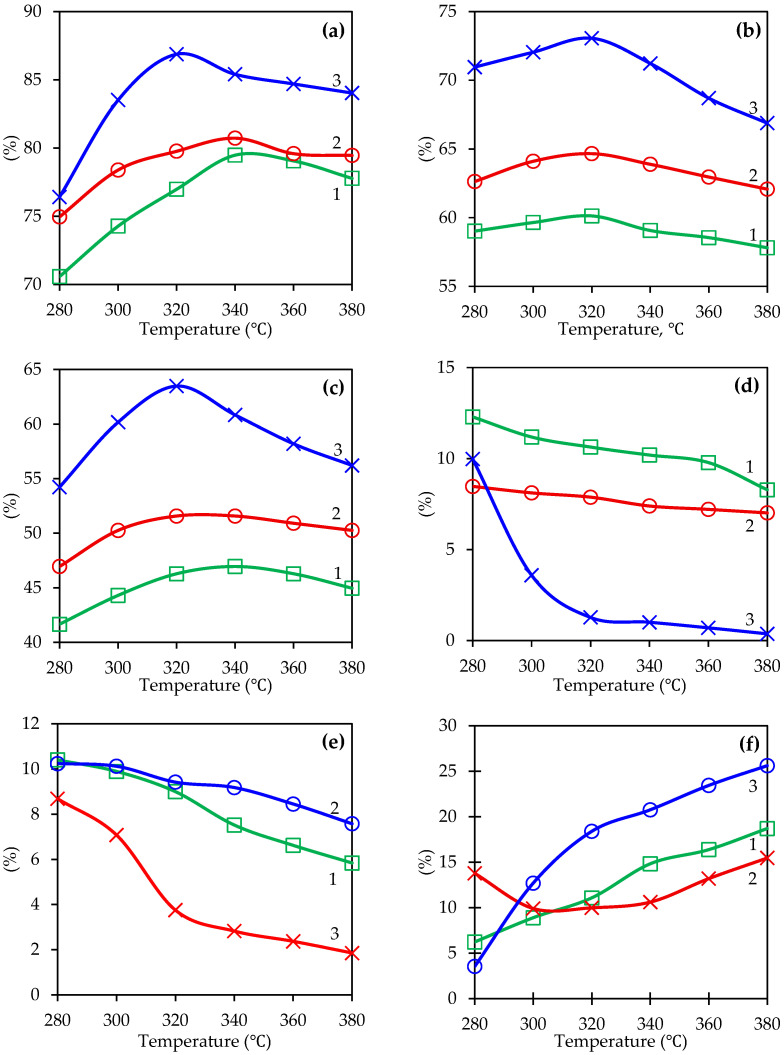
Temperature dependencies of the catalytic activity of 4-methylpyridine oxidation on various catalysts: (1) V-Cr; (2) V-Ti; (3) V-Ti-Cr; (**a**) 4-methylpyridine conversion; (**b**) isonicotinic acid selectivity; (**c**) isonicotinic acid yield; (**d**) pyridine-4-aldehyde yield; (**e**) pyridine yield; (**f**) CO_2_ output.

V-Cr, V-Ti and V-Ti-Cr catalysts were investigated for the 4-methylpyridine oxidation, with a mole ratio of 4-methylpyridine:O_2_:H_2_O = 1:10.48:76.87 conditions. The corresponding catalytic performance results are shown in Figure 6. Analysis of the dependence of 4-methylpyridine conversion, selectivity of isonicotinic acid formation, and its yield (Figure 6c) on temperature in the range of 280–380 °C shows that for all catalysts studied, there is a clear temperature dependence, where it first increases and then decreases again after reaching a maximum. This pattern can be associated with the activation of surface oxygen centres of catalysts. As can be seen from Figure 6a, the conversion of methylpyridine on the V-Ti-Cr catalyst reaches 86.88% at 320 °C. However, only 80.73% and 79.48% conversion of methylpyridine is achieved for the V-Ti and V-Cr catalysts at 340 °C. At the same time, the selectivity of isonicotinic acid formation (Figure 6b) at 320 °C is 73.06% on the V-Ti-Cr catalyst, while for V-Ti it is 64.66% and for V-Cr it is 64.13% under the same conditions.

The superior catalytic properties of the V-Ti-Cr catalyst over V-Cr and V-Ti are fully consistent with its better morphological characteristics. BET analysis indicates high texture values of 57.59 m^2^/g and a pore volume of 0.0353 cm^3^/g, which ensure maximum accessibility of active centres. X-ray diffraction analysis confirms the formation of thermally stable mixed V-Cr-Ti oxides without large crystallites, and thermogravimetric analysis/differential thermal analysis shows the mildest thermal change among all samples and minimal sintering under the same test conditions. The synergy of Ti and Cr enhances reversible oxygen transfer and stabilises V^5+^ centres, which explains such a significant increase in the degree of transformation even at moderate temperatures [53]. However, this combination makes the surface sensitive to ‘oxygen supersaturation’ at elevated temperatures, which leads to an increase in the proportion of secondary deep oxidation pathways and, as a result, to a decrease in selectivity above 320 °C.

It is noticeable that the behaviour dynamics for V-Cr and V-Ti systems are moderate. For V-Cr, this is explained by the previously established participation of Cr^6+^/Cr^3+^ pairs in reversible oxygen transfer, which provides controlled oxidative capacity without a tendency to overload the surface with oxygen. For V-Ti, this is explained by the dispersion of V^5+^ fragments on the titanium carrier. With increasing temperature, the proportion of easily formed intermediate organic products (4-pyridinecarbaldehyde and pyridine) (Figure 6d,e) tends to decrease while the portion of the deep oxidation product CO_2_ increases (Figure 6f). For 4-pyridinecarbaldehyde, a tendency towards a decrease in yield with increasing temperature was observed, but at different rates and degrees for different catalysts. In the V-Ti-Cr system, the aldehyde content decreases sharply from 9.97% to 1.27% at 320 °C, and then almost completely disappears at 380 °C to 0.37%.

**Figure 7:**
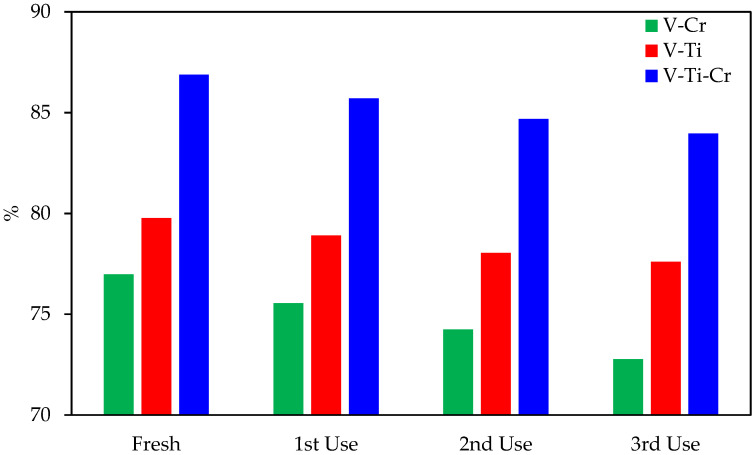
Catalytic stability of V-Ti, V-Cr and V-Ti-Cr catalysts during reuse.

To evaluate the catalytic stability of the synthesised catalysts in the oxidation of 4-methylpyridine, experiments were conducted on their reuse under identical reaction conditions (320 °C). The corresponding results are shown in Figure 7. After each catalytic cycle, the catalyst was separated by filtration, washed with ethanol, dried at 80 °C, and reused in the next cycle. It was found that the V-Ti catalyst showed higher catalytic stability compared to chromium-containing catalysts. The conversion of 4-methylpyridine in the 1st, 2nd, and 3rd recycling was 78.90%, 78.04%, and 77.60%, respectively, for V-Ti, indicating only a slight decrease in activity. At the same time, a significant decrease in conversion was observed for the V-Cr and V-Ti-Cr catalysts after each cycle. Thus, for the V-Cr catalyst, the conversion of 4-methylpyridine decreased from an initial 76.98% to 72.77%, while for the V-Ti-Cr catalyst, it decreased from 86.88% to 83.24%. This may be due to the increased oxidative capacity of chromium. It is known that chromium-containing catalysts promote deeper oxidation of organic compounds with the formation of CO_2_. In our case, it was found that with the V-Cr catalyst, the CO_2_ yield was 11.07%, while with the V-Ti-Cr catalyst, it reached 18.38%. These values are the highest among the catalytic systems studied.

## 3. Materials and Methods

### 3.1. Materials

In this study, analytical-grade chemical reagents were used as starting materials without additional purification: vanadyl sulphate trihydrate (VOSO_4_·3H_2_O), chromium chloride hexahydrate (III) hexahydrate (CrCl_3_·6H_2_O), and a solution of titanium (III) chloride with a mass fraction of TiCl_3_ of 10–15% to obtain the corresponding catalytic systems. Citric acid (C_6_H_8_O7) was used as a complexing agent, and high-purity ethylene glycol (C_2_H_6_O_2_) (about 97%) was used as a polycondensation reagent.

### 3.2. Catalyst Preparation

The V-Cr, V-Ti and V-Ti-Cr catalysts are synthesised using the Pechini method. In total, 10 moles of citric acid were dissolved in 100 mL of distilled water in a 500 mL beaker at 70 °C with constant stirring until complete dissolution. The amount of vanadium precursor VOSO_4_·3H_2_O corresponding to 1 mole of vanadium was added to the resulting solution with continuous stirring. Next, each catalyst was prepared by adding the appropriate precursors in an amount sufficient to provide 1 mole of the active component: for V-Cr—CrCl_3_·6H_2_O, for V-Ti—titanium (III) chloride solution (10–15%) TiCl_3_, and for V-Ti-Cr—both CrCl_3_·6H_2_O and TiCl_3_. The mixture was thoroughly stirred until all components were completely dissolved and a homogeneous reaction system was formed. Ethylene glycol was then added to the solution in a molar ratio of 1:1 relative to citric acid (10 moles). The mixture was heated to 90 °C to remove excess water and form a polymer resin. The resulting resin was dried at 120 °C for 12 h. The dried material was heated at 400 °C for 4 h to remove organic components, after which the resulting material was ground and mixed with distilled water. This mass was extruded through a syringe, after which the extrudates were dried and finally calcined at 800 °C for 4 h. The calcined extrudates were mechanically crushed (cut) and sieved to isolate the 0.25–0.5 mm fraction, which was used in catalytic tests. The fine fraction (powder) of the same extrudates was used for physicochemical analyses (XRD, etc.). This guarantees the complete identity of the phase and chemical composition of the catalyst in the reactor and the samples subjected to instrumental research methods.

### 3.3. Apparatus and Experimental Procedure

**Figure 8:**
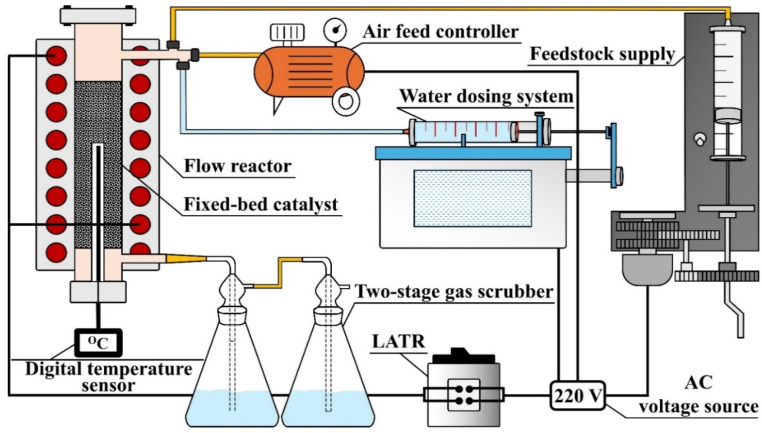
Apparatus for the catalytic oxidation of 4-methylpyridine.

Experimental studies of gas-phase catalytic oxidation of 4-methylpyridine were carried out in a flow reactor (Figure 8) with a fixed catalyst layer 10 cm^3^ thick. The reactor was heated by an electric furnace connected via a laboratory autotransformer (LATR). The temperature was controlled using a thermocouple and a digital temperature sensor. The catalyst in granular form, 3–5 mm in size and with a total mass of 10 g, was loaded into a removable cylindrical metal insert with an inner diameter of 16 mm and a layer height of 40 mm. Air was supplied using a flow regulator. Water vapour was dosed through a water supply system comprising a syringe pump and an evaporator. 4-methylpyridine was fed into the reactor through a separate dosing pump. The reaction products were captured in a two-stage absorption scrubber filled with water.

### 3.4. Research Methods

#### 3.4.1. X-Ray Diffraction

The diffractograms of the catalysts were recorded by XRD using a Dandong Aolong Radiative Instrument Group Co., Ltd. (Dandong, China) device. The recording was performed at X-ray tube voltage of 40 kV and a current of 40 mA. To improve the resolution, a slit system with parameters of 1° × 1° × 0.2 mm was used, as well as a monochromator. The scanning range was from 5 to 80° at 2θ. The diffractograms were processed using the Materials Data software package (https://www.materialsdata.com/) (Materials Data, Inc., Livermore, CA, USA). The Peak ID Extended Report algorithm was used to select peaks with the following settings: 19-point filter (parabolic filter), threshold value 3.0, cutoff 0.1%, background correction (BG) = 3/1.0. The maxima were determined using the Summit method.

#### 3.4.2. Raman Spectroscopy

Raman spectroscopy was used to study the structural characteristics of catalysts synthesised by the Pechini method. The measurements were performed on a Solver Spectrum Raman spectrometer (NT-MDT, Zelenograd, Russia) using blue laser excitation with a wavelength of 473 nm at a power of 35 mW. Neutral light filters (ND = 0, ND = 0.5, and ND = 1) were used to prevent overheating of the samples. A 600/600 diffraction grating was used as the dispersing element. The spectra were recorded in the range from 103 to 3200 cm^−1^ with a maximum number of counts to 2300.

#### 3.4.3. Thermogravimetric Analysis

Thermogravimetric analysis was used to evaluate the thermal stability and decomposition characteristics of the catalysts under study. Measurements were performed on an SKZ1053 (SKZ Industrial Co., Limited, Jinan, China) thermogravimetric analyser in the temperature range of 30–400 °C. Sample weights of 2–4 mg were placed in a 70 μL platinum crucible. The experiments were performed in an inert nitrogen atmosphere at a gas flow rate of 50 mL/min and a heating rate of 35 °C/min. The heating rate of 35 °C/min was selected to enhance the sensitivity of the DTA signal and clearly identify low-energy thermal events that are poorly resolved at lower heating rates. For a more detailed interpretation of the thermogravimetric curves, DTA curves were additionally calculated, reflecting the temperature differences between the sample and a reference under the same heating conditions. Joint analysis of TGA and DTA made it possible not only to determine the magnitude and sequence of mass loss stages, but also the identification of the temperature intervals corresponding to the most intense endothermic or exothermic processes.

#### 3.4.4. Brunauer–Emmett–Teller Method

The textural characteristics of the catalysts were determined by low-temperature nitrogen adsorption–desorption at 77 K using an ASAP 2400 Micrometrics Gemini VII 2390a instrument (Micromeritics Instrument Corporation, Norcross, GA, USA). Nitrogen (N_2_) was used as an adsorbent. The sample weights ranged from 0.15 to 0.20 g. Before measurement, the samples were degassed under vacuum at a rate of 1000 mm Hg/min. The equilibrium time at each point of the isotherm was 5 s. The density of the sample was taken to be 1.000 g/cm^3^. Isotherms were recorded in the relative pressure range P/P_0_ = 0.01–0.99. The specific surface area was calculated using the BET method. The pore size distribution and total pore volume were determined using the BJH method. The contribution of micropores was estimated using the t-Plot model. All calculations were performed using the built-in Gemini VII Version 5.03 software.

#### 3.4.5. Scanning Electron Microscopy

The morphology of catalysts synthesised using the Pechini method was studied using a Quanta 200i 3D SEM scanning electron microscope (FEI Company, Hillsboro, OR, USA) in low vacuum mode. The images were recorded at an accelerating voltage of 15.0 kV, a magnification of 1000 times, a working distance of 12.4 mm, and an image field width of 298 μm. The pressure range in low vacuum mode was 10–130 Pa (0.1–1.0 torr), which allowed studies to be conducted without additional application of a conductive coating and ensured the preservation of the original surface structure of the catalysts. The accelerating voltage ranged from 200 V to 30 kV, which made it possible to analyse both the micro-relief of the surface and to identify morphological features at different scales. In addition, elemental composition and distribution of the catalysts were analysed using energy-dispersive X-ray spectroscopy (EDX) coupled to the SEM, providing complementary information on the presence and relative distribution of V, Cr, and Ti in the synthesised materials.

#### 3.4.6. ICP-OES Analysis

The chemical composition of the catalysts was determined using an inductively coupled plasma atomic emission spectrometer ICP-OES, model 8300 DV (PerkinElmer Inc., Shelton, CT, USA). The method is based on acid dissolution of samples and subsequent atomic emission analysis. Three 0.1 g weights were used for each catalyst. The weight was placed in a porcelain crucible and fired in a muffle furnace at 600 °C for 2 h. The contents of the crucible were transferred to a 250 cm^3^ conical flask and washed with a mixture of hydrochloric and nitric acids (3:1). Thirty cm^3^ of the same acid was added to the mixture and boiled under a watch glass for 20–25 min. The solution was evaporated to a volume of 1–2 cm^3^, cooled and transferred to a 100 cm^3^ volumetric flask. The resulting solution was sprayed into an air-argon ICP-OES flame to measure the mass fraction of metals. Each solution was analysed at least three times, and the arithmetic mean value was taken for calculation.

#### 3.4.7. Gas Chromatographic Analysis

Chromatographic analysis of oxidation products of 4-methylpyridine was carried out by chromatograph ‘Cvet-106’ with flame ionisation detector. The gas carrier was argon. The glass column, 1000 mm long and with a diameter of 4 mm, is filled with a stationary liquid phase of 25% polyethylene adipate + 2% H_3_PO_4_ on the INZ-600 diatomite carrier. The column thermostat temperature is 125 °C, and the evaporator temperature is 200 °C. As the internal standard, we used 2,4,6-kollidin. For sample preparation, 2 mL of ammonia (NH_3_) solution and 1 mL of internal standard (2,4,6-kollidin) were added to a 25 mL volumetric flask, after which the volume was brought to the mark with a working solution (WS) of liquid products. The resulting mixture was thoroughly mixed until the components were evenly distributed. Using a microsyringe, 4 μL was taken from the prepared mixture and injected into a gas chromatograph. Each measurement was performed three times by sequentially injecting the sample from the same prepared flask to assess accuracy and reproducibility.

#### 3.4.8. Titration

The content of isonicotinic acid in the reaction products was determined by acid-base titration. For analysis, a 10 mL aliquot was taken from working solution and titrated with 0.00497 N potassium hydroxide (KOH) solution. Phenolphthalein (2–4 drops) was used as an indicator, and the equivalence point was controlled using a pH-150MI pH metre (OOO ‘Izmeritelnaya Tekhnika’, Moscow, Russia). To ensure the accuracy and reproducibility of the results, each titration was performed three times so that the discrepancy between parallel measurements did not exceed ±0.1 mL of titrant consumed, after which the average value was calculated.

## 4. Conclusions

Based on the results of this study, several conclusions can be drawn. It has been established that catalytic results are directly related to the structural, textural, and thermal characteristics of catalysts. The optimal process temperature is around 320 °C, where the combination of 4-methylpyridine conversion and isonicotinic acid formation selectivity is most favourable for all catalysts studied. At the same time, above 320 °C, the activity of all catalysts decreases due to the intensification of deep oxidation reactions and an increase in CO_2_ yield caused by supersaturation of the surface with active oxygen. The large surface area according to the BET method and developed porosity (V-Ti-Cr) increase the probability of adsorption and uniform distribution of the reagent over the active surface, reducing local overload and promoting the sequential conversion of intermediate products to INA. This results in maximum conversion (86.88%) and selectivity (73.06%) values compared to binary V-Cr and V-Ti systems. Raman indicators of the transition from polymerized vanadium structures (terminal V=O and bridging V-O-V bonds) to isolated and mixed vanadate complexes (Cr-O and V-O-Ti) are a predictive marker of the trend towards favourable sequential selectivity.

The coincidence of thermoanalytical and Raman data confirms that the stabilisation of polyvanadate structures and the formation of dispersed CrVO_4_-type clusters in the V-Ti-Cr system are completed in the range of 400–600 °C, ensuring high catalytic stability. A sharp decrease in mass at temperatures above 350 °C for V-Cr and V-Ti indicates the development of phase transformations and the formation of mixed oxides, which is confirmed by the appearance of characteristic Raman modes V=O and bridge bonds V-O-Cr or V-O-Ti, as well as by X-ray diffraction analysis data.

Prospects for further research in this area include theoretical calculations using density functional theory (DFT) to study in detail the nature of active centres and the mechanisms of 4-methylpyridine adsorption on the surfaces of V-Cr-Ti catalysts. In addition, we plan to study the processes of molecular oxygen activation and the energy barriers of the reaction stages, which will allow us to justify the observed differences in the catalytic activity of the studied systems at the molecular level. Another important area of future work will be to study the stability of catalysts under long-term resource testing conditions.

## Figures and Tables

**Table 1:** Preliminary assignment of bands for catalytic systems under Raman excitation.

Assignment	Wavenumber, cm^−1^			Reference
	V-Cr	V-Ti	V-Ti-Cr	
T_z_ translation			104	[46]
B_1_g—lattice vibration		140	148	[48,49]
V-O bending/lattice rotation	140	140	148	[46,47]
O-V-O bending	283		286	[46,47]
V-O bending/internal stretch		315		[46]
Skeleton bending/V-O mode	405		405	[46]
ridging V-O-V bonds			480	[46]
B1g + Ag modes	516			[49]
bridging O-V-O bending/stretch			526	[46]
V-O stretching (internal)	688		699	[46,47]
network stretching/V-O species		840–868		[46,50]
symmetric CrO stretching	990			[51]
V=O terminal stretching	990	840–868	994	[46,47]

**Table 2:** BET surface area and pore properties of oxide catalysts.

Catalysts	BET Surface Area, m^2^/g	BJH Surface Area, m^2^/g	BJH Pore Volume, cm^3^/g
V-Cr	1.3780	0.9437	0.000678
V-Ti	2.0470	0.7539	0.000384
V-Ti-Cr	57.5891	66.4401	0.035257

**Table 3:** ICP-OES results of catalysts.

	V		Cr		Ti	
Catalysts	Nominal	Actual	Nominal	Actual	Nominal	Actual
V-Cr	1	1	1	0.9	-	-
V-Ti	1	1	-	-	1	0.882
V-Ti-Cr	1	1	1	0.875	1	0.931

## Data Availability

The data presented in this study are available on request from the corresponding authors.

## Abbreviations

The following abbreviations are used in this manuscript:

4-MP	4-methylpyridine
XRD	X-ray diffraction
TGA	Thermogravimetric analysis
DTA	Differential thermal analysis
BET	Brunauer–Emmett–Teller
BJH	Barrett–Joyner–Halenda
SEM	Scanning Electron Microscopy
TB	Tuberculosis
WHO	World Health Organization
INH	Isonicotinic acid hydrazide
INA	Isonicotinic acid
WS	Working solution

## References

[1] Tuberculosis. World Health Organization 2025. https://www.who.int/news-room/fact-sheets/detail/tuberculosis#:~:text=,TB%20is%20curable%20and%20preventable.

[2] Luies L., du Preez I. (2020). The Echo of Pulmonary Tuberculosis: Mechanisms of Clinical Symptoms and Other Disease-Induced Systemic Complications. Clin. Microbiol. Rev..

[3] Tobin E.H., Tristram D. Tuberculosis Overview. StatPearls 2025. https://www.ncbi.nlm.nih.gov/books/NBK441916/?utm_source=chatgpt.com.

[4] Field S.K., Escalante P., Fisher D.A., Ireland B., Irwin R.S. (2018). Cough Due to TB and Other Chronic Infections: CHEST Guideline and Expert Panel Report. Chest.

[5] Smiljić S., Radović B., Ilić A., Trajković G., Savić S., Milanović Z., Mijović M. (2019). Differences and Similarities between the Symptoms and Clinical Signs in Patients with Pulmonary Tuberculosis and Pneumonia. Vojnosanit. Pregl..

[6] (2023). Module 4: Cardiopulmonary Conditions. Package of Interventions for Rehabilitation.

[7] Tanimura T., Jaramillo E., Weil D., Raviglione M., Lönnroth K. (2014). Financial burden for tuberculosis patients in low- and middle-income countries: A systematic review. Eur. Respir. J..

[8] Cardinale L., Parlatano D., Boccuzzi F., Onoscuri M., Volpicelli G., Veltri A. (2015). The Imaging Spectrum of Pulmonary Tuberculosis. Acta Radiol..

[9] Chen Z., Wang T., Du J., Sun L., Wang G., Ni R., An Y., Fan X., Li Y., Guo R. (2025). Decoding the WHO Global Tuberculosis Report 2024: A Critical Analysis of Global and Chinese Key Data. Zoonoses.

[10] Sahra S., Agudelo Higuita N.I., Alkozah M. Tuberculosis (TB) Treatment & Management. Medscape 2024. https://emedicine.medscape.com/article/230802-treatment?form=fpf.

[11] Saukkonen J.J., Duarte R., Munsiff S.S., Winston C.A., Mammen M.J., Abubakar I., Acuña-Villaorduña C., Barry P.M., Bastos M.L., Carr W. (2025). Updates on the Treatment of Drug-Susceptible and Drug-Resistant Tuberculosis: An Official ATS/CDC/ERS/IDSA Clinical Practice Guideline. Am. J. Respir. Crit. Care Med..

[12] Davies G.R. (2024). Reconsidering the Role of Isoniazid in Drug-Resistant Tuberculosis. Am. J. Respir. Crit. Care Med..

[13] Nikolova-Mladenova B., Momekov G., Zhivkova Z., Doytchinova I. (2023). Design, Synthesis and Cytotoxic Activity of Novel Salicylaldehyde Hydrazones against Leukemia and Breast Cancer. Int. J. Mol. Sci..

[14] Shimizu S., Watanabe N., Kataoka T., Shoji T., Abe N., Morishita S., Ichimura H. (2007). Ullmann’s Encyclopedia of Industrial Chemistry.

[15] Vereshchagin L.I., Kotlyarevskii I.L. (1961). Advances in the Synthesis of Alkyl Pyridines. Russ. Chem. Rev..

[16] Jubilant Life Sciences Ltd (2011). Process for Producing Pyridine Carboxylic Acids. U.S. Patent.

[17] Chuck R. (2005). Technology Development in Nicotinate Production. Appl. Catal. A Gen..

[18] Lonza Electric and Chemical Works Ltd (1956). Process and Apparatus for the Preparation of Pyridine Carboxylic Acids. GB Patent.

[19] Lisicki D., Nowak K., Orlińska B. (2022). Methods to Produce Nicotinic Acid with Potential Industrial Applications. Materials.

[20] Kadirbekov K., Buzayev N., Tussupkaliyev Y., Oshakbayev M. (2025). Oxidation of 4-Methylpyridine on Vanadium-Based Catalysts Modified with Titanium and Manganese. Catalysts.

[21] Miao Y.C., Zhai Z.B., Yang S.C., Wang J.Q. (2011). Catalytic Oxidation of 4-Methyl Pyridine to Isonicotinic Acid by H_2_O_2_ over Co-MCM-41. Chin. J. Appl. Chem..

[22] Vorob’ev P.B., Gabdrakipov V.Z., Mikhailovskaya T.P., Sembaev D.K. (2001). Reactivity of Isomeric Picolines in Oxidative Ammonolysis on a Vanadium Oxide Catalyst. Russ. J. Gen. Chem..

[23] Vorobyev P., Mikhailovskaya T., Yugay O., Serebryanskaya A., Chukhno N., Imangazy A. (2018). Catalytic Oxidation of 4-Methylpyridine on Modified Vanadium Oxide Catalysts. Iran. J. Chem. Chem. Eng..

[24] Vorobyev P.B., Serebryanskaya A.P. (2019). Reactivity of Selected Mono- and Dimethylpyridines under Conditions of Oxidative Ammonolysis. Russ. J. Gen. Chem..

[25] Vorobyev P., Serebryanskaya A., Yugay O., Mikhailovskaya T. (2020). Oxidative Ammonolysis of 3,4-Dimethylpyridine on Vanadium Oxide Catalysts. J. Serb. Chem. Soc..

[26] Chesalov Y.A., Andrushkevich T.V., Baltakhinov V.P. (2016). FTIR Study of the Role of Surface Complexes in a Transformation of Picoline Isomers on Vanadium–Titanium Oxide Catalysts. Vibr. Spectrosc..

[27] Vorobyev V.B., Mikhaylovskaya T.P., Yugai O.K., Serebryanskaya A.P., Kurmakyzy R. (2019). Vapour-Phase Oxidation of 3- and 4-Methylpyridines on Vanadium Oxide Catalysts Modified by Titanium and Chromium Oxides. Chem. J. Kaz..

[28] Yugay O.K., Mikhailovskaya T.P., Sembaev D.K., Vorobyev P.B. (2012). Oxidation of 3- and 4-Methylpyridines on Vanadium-Anatase and Vanadium-Rutile Catalysts. Eurasian Chem. Technol. J..

[29] Buzayev N.A., Kadirbekov K.A., Tolemisova D.K., Basbayeva G.S. (2024). Oxidation of 4-Methylpyridine on V–Cr–O Catalyst. Chem. J. Kaz..

[30] Yang G.J., Huang H.F., Lu H.F., Liu H.Y., Chen Y.F. (2007). Oxidation of 4-Picoline to Isonicotinic Acid on V-Ti-O Catalysts. J. Chem. Eng. Chin. Univ..

[31] Vorobyev P.B., Mikhailovskaya T.P., Tolemisova D.K. (2019). Oxidative Transformation of 2-, 3- and 4-Methylpyridines in the Presence of Vanadium Oxide Catalyst Modified by Titanium and Aluminum Oxides. Chem. J. Kaz..

[32] Vorobyev P.B., Saurambaeva L.I., Mikhailovskaya T.P. (2013). Oxidation of 3- and 4-Methylpyridines on Modified Vanadium Oxide Catalysts. Russ. J. Gen. Chem..

[33] Mikhailovskaya T.P., Vorobyev P.B., Kadirbekov K.A., Kurmakyzy R. (2020). Catalytic Activity of Vanadium Oxide Catalysts Modified by Ti, Sn, Zr Oxides in the Partial Oxidation of 4-Methylpyridine. Chem. J. Kaz..

[34] Vorobyev P.B., Serebryanskaya A.P. (2012). Oxidative Ammonolysis of 3(4)-Methyl- and 3,4-Dimethylpyridines Using Vanadium Oxide Catalysts. Russ. J. Gen. Chem..

[35] Mikhailovskaya T.P., Yugay O.K., Chukhno N.I., Sembaev D.K. (2012). Oxidative Ammonolysis of 3- and 4-Methylpyridines on Vanadium Oxide Catalysts Modified with Titanium and Tin Oxides. Russ. J. Appl. Chem..

[36] Ye L., Ma J., Zhang J., Yin W., Xie K. (2023). Insight into the Role and Evidence of Oxygen Vacancies in Porous Single-Crystalline Oxide to Enhance Catalytic Activity and Durability. Research.

[37] Luo D., Luo R., Wang X., Chang X., Yang T., Chen S., Zhao Z.-J., Gong J. (2025). Role and Regulation of Surface Oxygen Vacancies in Vanadium-Based Oxides for Chemical Looping Oxidative Dehydrogenation of Propane. Chem. Sci..

[38] Kandage M.M., Marszewski M. (2024). On the Synthesis and Formability of High-Entropy Oxides. J. Mater. Sci..

[39] Sunde T.O.L., Grande T., Einarsrud M.A., Klein L., Aparicio M., Jitianu A. (2016). Modified Pechini Synthesis of Oxide Powders and Thin Films. Handbook of Sol-Gel Science and Technology.

[40] Vargas-Urbano M.A., Marín L., Castillo W.M., Rodríguez L.A., Magén C., Manotas-Albor M., Diosa J.E., Gross K. (2022). Effect of Ethylene Glycol:Citric Acid Molar Ratio and pH on the Morphology, Vibrational, Optical and Electronic Properties of TiO_2_ and CuO Powders Synthesized by Pechini Method. Materials.

[41] Basha M.T., Abou-Krisha M.M., Saad F.A., Shah R.K., Abdelrahman E.A. (2025). Pechini Derived Multifunctional MgO-Based Chromate Nanocomposites for Superior Brilliant Green Dye Adsorption. Sci. Rep..

[42] Nassar M.Y., Ali A.A., Amin A.S. (2017). A Facile Pechini Sol–Gel Synthesis of TiO_2_/Zn_2_TiO_4_/ZnO/C Nanocomposite: An Efficient Catalyst for the Photocatalytic Degradation of Orange G Textile Dye. RSC Adv..

[43] Pryston D.B.d.A., Martins T.V. dos S., Vasconcelos Júnior J.A., Avelino D.O. da S., Meneghetti M.R., Meneghetti S.M.P. (2023). Investigation of CeO_2_, MoO_3_, and Ce_2_(MoO_4_)_3_ Synthesized by the Pechini Method as Catalysts for Fructose Conversion. Catalysts.

[44] Martins T.V.d.S., Pryston D.B.d.A., Meneghetti S.M.P., Meneghetti M.R. (2023). Influence of Synthesis Methodology on the Properties and Catalytic Performance of Tin, Niobium, and Tin–Niobium Oxides in Fructose Conversion. Catalysts.

[45] Ramos-Alvarez P., Villafuerte-Castrejón M.E., González G., Cassir M., Flores-Morales C., Chávez-Carvayar J.A. (2017). Ceria-Based Electrolytes with High Surface Area and Improved Conductivity for Intermediate Temperature Solid Oxide Fuel Cells. J. Mater. Sci..

[46] Wang Y., Rosowski F., Schlögl R., Trunschke A. (2022). Oxygen Exchange on Vanadium Pentoxide. J. Phys. Chem. C..

[47] Mouratis K., Tudose V., Romanitan C., Pachiu C., Tutunaru O., Suchea M., Couris S., Vernardou D., Emmanouel K. (2020). Electrochromic Performance of V2O5 Thin Films Grown by Spray Pyrolysis. Materials.

[48] Gupta S.K., Desai R., Jha P.K., Sahoo S., Kirin D. (2009). Titanium Dioxide Synthesized Using Titanium Chloride: Size Effect Study Using Raman Spectroscopy and Photoluminescence. J. Raman Spectrosc..

[49] Belekbir S., El Azzouzi M., El Hamidi A., Rodríguez-Lorenzo L., Santaballa J.A., Canle M. (2020). Improved Photocatalyzed Degradation of Phenol, as a Model Pollutant, over Metal-Impregnated Nanosized TiO_2_. Nanomaterials.

[50] Aruchamy K., Sudarsan D., Ajith M., Sreekumar A.A., Ayyasamy U.M., Manickam S. (2024). Enhanced photocatalytic activity of V3O7/V2O5—Reduced graphene oxide nanocomposite towards methylene blue dye degradation. Environ. Sci. Pollut. Res..

[51] Dines T.J., Inglis S. (2003). Raman Spectroscopic Study of Supported Chromium(VI) Oxide Catalysts. Phys. Chem. Chem. Phys..

[52] Koivikko N. (2023). Silica-Titania Supported Vanadia Catalysts in the Utilization of Industrial Sulfur-Contaminated Gaseous Methanol Streams. Acta Univ. Oul..

[53] Jin Y., Li J., Fan K., Chen Y., Yang Y., Liu X. (2024). The Nature of Synergy Effects between VOx and TiO_2_ in Low Temperature NH3-SCR Reaction. J. Environ. Chem. Eng..

